# Rapid Preparation of a Large Sulfated Metabolite Library for Structure Validation in Human Samples

**DOI:** 10.3390/metabo10100415

**Published:** 2020-10-16

**Authors:** Mario S. P. Correia, Weifeng Lin, Arash J. Aria, Abhishek Jain, Daniel Globisch

**Affiliations:** Department of Medicinal Chemistry, Science for Life Laboratory, Uppsala University, Box 574, SE-75123 Uppsala, Sweden; Mario.correia@ilk.uu.se (M.S.P.C.); Weifeng.Lin@ilk.uu.se (W.L.); arash.j.aria@gmail.com (A.J.A.); Abhishek.Jain@ilk.uu.se (A.J.)

**Keywords:** sulfated metabolites, metabolomics, structure validation, chemical synthesis, phase II metabolism, microbiome

## Abstract

Metabolomics analysis of biological samples is widely applied in medical and natural sciences. Assigning the correct chemical structure in the metabolite identification process is required to draw the correct biological conclusions and still remains a major challenge in this research field. Several metabolite tandem mass spectrometry (MS/MS) fragmentation spectra libraries have been developed that are either based on computational methods or authentic libraries. These libraries are limited due to the high number of structurally diverse metabolites, low commercial availability of these compounds, and the increasing number of newly discovered metabolites. Phase II modification of xenobiotics is a compound class that is underrepresented in these databases despite their importance in diet, drug, or microbiome metabolism. The *O*-sulfated metabolites have been described as a signature for the co-metabolism of bacteria and their human host. Herein, we have developed a straightforward chemical synthesis method for rapid preparation of sulfated metabolite standards to obtain mass spectrometric fragmentation pattern and retention time information. We report the preparation of 38 *O*-sulfated alcohols and phenols for the determination of their MS/MS fragmentation pattern and chromatographic properties. Many of these metabolites are regioisomers that cannot be distinguished solely by their fragmentation pattern. We demonstrate that the versatility of this method is comparable to standard chemical synthesis. This comprehensive metabolite library can be applied for co-injection experiments to validate metabolites in different human sample types to explore microbiota-host co-metabolism, xenobiotic, and diet metabolism.

## 1. Introduction

Metabolomics is the most recent major “omics” research field and is in an ongoing process for optimization of data quality, sample preparation, and the development of bioinformatic tools [1,2,3]. Metabolites in any biological sample are of high structural complexity, different polarity, and have concentration differences of several orders of magnitude, which is in stark contrast to the other three major omics fields (genomics, transcriptomics, and proteomics) with defined sets of natural building blocks. Extracted metabolite mixtures are commonly analyzed by the following two standard detection methods: (i) nuclear magnetic resonance spectroscopy and (ii) chromatographic separation systems (e.g., gas or liquid phase) coupled to mass spectrometry [4,5]. Mass spectrometry has evolved as the dominant method in metabolomics and identification of a metabolite requires chromatographic information and the mass-to-charge ratio (*m/z*) for determination of the correct chemical structure. The structure validation process is currently considered to be one of the major bottlenecks in metabolomics analysis.

The development of robust authentic metabolite databases for rapid and automated metabolite structure validation is not a trivial task due to the structural complexity and large number of metabolites. Powerful and sophisticated metabolite databases have been developed such as GNPS, HMDB, MoNa, SIRIUS, NIST, and METLIN [6,7,8,9,10,11,12]. These constantly increasing experimental and computational standard libraries contain large sets of metabolite fragmentation pattern and serve as important tools for the characterization of metabolites in any sample type. However, new tools and advanced analyses lead to the identification of yet unidentified and uncharacterized metabolites that are not present in these databases and not directly available for the scientific community [13,14]. The structure of validated metabolites is reported at different levels of confidence and the highest level requires authentic chemical standards [15]. These standards are required to distinguish regioisomers that are metabolites with the same chemical formula and *m/z* value, which can in most cases not be distinguished based on their fragmentation pattern only [15,16]. For example, hydroxybenzoic acid can have three different substitution pattern with the phenolic alcohol either in ortho, meta or para position to the carboxylic acid. These three regioisomers have different chemical and physical properties and are products of different biochemical pathways [17,18]. Therefore, the correct stereochemical information for the identified compound is required to draw the correct biochemical conclusions in metabolomics studies. An authentic standard is required to validate the metabolite structure and determine the specific characteristics such as the retention time, the *m/z* value, and the MS/MS fragmentation pattern. These synthetic standards are utilized in co-injection experiments for direct comparison with the natural metabolite.

We have recently developed new chemical biology tools for the discovery of unknown metabolites in human samples [19,20,21,22]. A metabolite class, of interest, is *O*-sulfated metabolites that have been widely linked with the co-metabolism between the human and its gut microbiome [19,20,23,24]. Sulfated compounds are commonly known to be part of the human phase II clearance process for a large percentage of xenobiotic compounds for excretion through urine or feces [25,26]. Indoxyl sulfate or *p*-cresyl sulfate are two examples for the metabolic interaction of mammalian and bacterial metabolism [19]. Despite the importance of this compound class, the number of these metabolite conjugates reported in the largest metabolite databases such as HMDB or METLIN is limited, and thus widely excluded in metabolomics studies. For investigation of this metabolite class, we recently developed a method combining enzymatic metabolite conversion with state-of-the-art metabolomics bioinformatic analysis [19,27]. Several previously unreported sulfated metabolites were discovered in human urine and fecal samples. Metabolite structures in these studies were validated at different confidence levels. Most assignments were performed by comparison of their mass spectrometric fragmentation pattern with databases, as commercially available sulfated compounds are scarce.

Due to the lack of available reference standards, we sought to prepare a comprehensive library of biologically relevant sulfated metabolites. As chemical synthesis of these compounds usually requires fully equipped organic chemistry infrastructure that is not available in many metabolomics laboratories, we have described a simple and straightforward method to quickly prepare a series of sulfated standards. This method can be used to efficiently produce a large number of reference compounds through parallel syntheses at the same quality as standard organic chemistry. We outline the preparation of a chemical library of 38 sulfated metabolites that are now available for validation in human samples.

## 2. Results and Discussion

**Figure 1:**
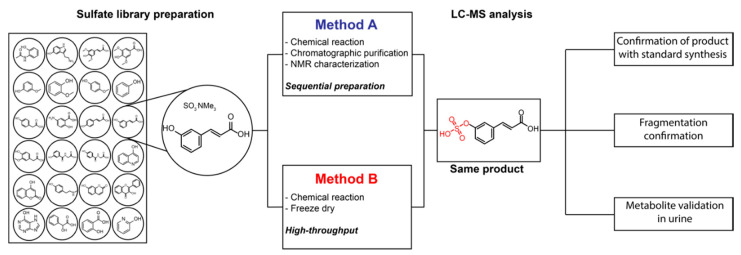
Workflow for the library preparation. Comparison between the obtained product from standard chemical synthesis (Method A) and the two-step preparation described in this manuscript (Method B).

In order to efficiently synthesize standard molecules for structure validation of sulfated metabolites for large-scale preparation of reference molecules, we sought to devise a straightforward synthetic strategy (Figure 1). Standard chemical synthesis protocols of metabolites usually require several chemical reactions including protecting group chemistry to avoid byproduct formation for most synthetic routes [28,29]. This is followed by preparative high-performance liquid chromatography (HPLC) purification and NMR characterization to confirm the chemical structure of the synthetic product (Method A) [19,30,31]. The purification of the crude product from a chemical reaction is time consuming and also requires specific equipment. In our developed procedure, reported herein (Method B), we are merely removing all reagents and the solvent after completed synthesis using a lyophilizer. Then, this product is reconstituted and analyzed via ultra-performance liquid chromatography coupled to mass spectrometry (UPLC-MS). This method can be used for large-scale and parallel preparation of new sulfated compounds. Herein, we detail a step-by-step evaluation of this method and provide validation of the quality for this synthetic procedure. The prepared standard compound library is available for rapid and efficient metabolite structure characterization in human samples. An additional advantage is the preparation scale at low quantities (0.2 mg) as the high sensitivity of the mass spectrometric analysis does not require large scale synthesis.

### 2.1. Validation of Standard Preparation Procedure

**Figure 2:**
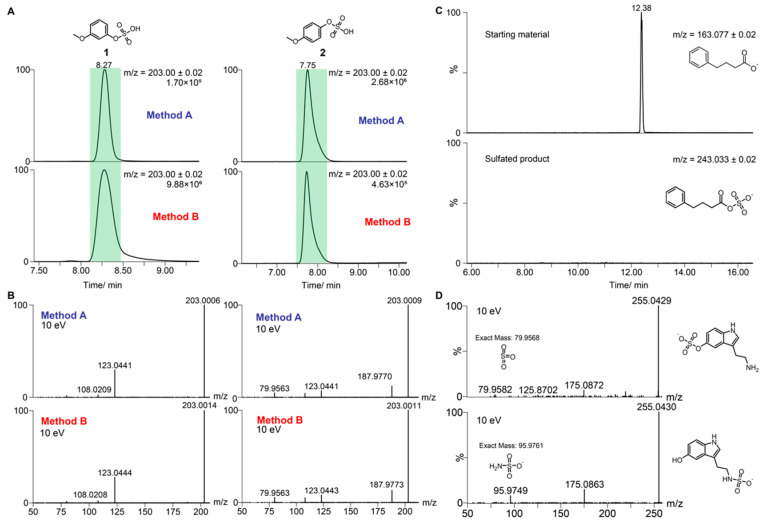
Comparison between the fully synthesized compounds and the prepared metabolites. (**A**) Injection of 3-methoxyphenol sulfate (**1**) and 4-methoxyphenol sulfate (**2**) synthesized using Method A (top) and Method B (bottom); (**B**) MS/MS fragmentation of 3-methoxyphenol sulfate (**1**) and 4-methoxyphenol sulfate (**2**) synthesized with Method A (top) and Method B (bottom) (10 eV); (**C**) Validation of the non-reactive carboxylic acids using this sulfation procedure for the test substrate phenylbutyric acid. Extracted ion chromatograms (EICs) validate no product formation after NMe_3_·SO_3_ treatment for 16 h; (**D**) MS/MS fragmentation comparison of *O*-sulfated serotonin (top) and *N*-sulfated serotonin (bottom) (10 eV).

In the first step, we validated our standard preparation strategy by comparing our method with metabolites prepared through standard chemical synthesis. The two regioisomers, 3-methoxyphenol sulfate (**1**) and 4-methoxyphenol sulfate (**2**), were first synthesized using the standard method (Method A). The two regioisomers are baseline separated and their MS/MS fragmentation result in almost identical fragmentation pattern (Figure 2A,B). On the basis of this similarity, these two isomers are a good example of metabolites that can only be distinguished in biological samples using authentic reference molecules. Then, each regioisomer **1** and **2** was prepared using Method B by mixing the sulfate reagent NMe_3_·SO_3_ (3 eq.), the corresponding alcohol or phenol (1 eq.), sodium hydroxide (1 eq.), and sodium bicarbonate (3 eq.). The solution was stirred for 16 h under inert gas conditions. The solvent of the reaction mixture was removed and re-dissolved to analyze the reaction using UPLC-MS analysis. Reference molecules prepared by either Method A or Method B have the same chromatographic properties and mass spectrometric fragmentation spectra (Figure 2A,B). This validates the applicability of Method B for construction of a sulfated metabolite library.

We next validated the regioselectivity of this reaction for Method B. While the method is easily applicable for sulfation of monohydroxylated compounds, many metabolites contain additional functionalities such as amines or carboxylic acids, which could lead to byproduct formation including bis-sulfation. In order to validate our method for these metabolites, we tested two different substrates. In the first reactivity analysis, we tested the sulfation reactivity and stability of carboxylic acids, which form labile sulfated compounds due to fast hydrolysis in acidic and neutral aqueous solutions. Phenylbutyric acid was chosen as a model substrate, which only contains one carboxylic acid as the reactive site. No consumption of the starting material was observed, and no formation of a sulfated product was detected (Figure 2C). On the basis of this observation, we can exclude sulfation of carboxylic acid functionalities. Furthermore, no carboxylic acid sulfation was observed for any substrate using Method B in the sulfate library construction. The second reactivity analysis was performed to test sulfation of primary amines. Serotonin was tested as an example substrate that contains both a primary amine and a 3-hydroxyindole functionality. Upon testing Method B for this compound, we obtained two different monosulfated products identified in the mass spectrometric analysis (Figure 2D). These two products were identified as 3-sulfohydroxyserotonin and *N*-sulfo-serotonin. No bis-sulfated product was identified in the reaction mixture. Mass spectrometric fragmentation experiments were performed to distinguish each structure based on their mass spectrometric fingerprint (Figure 2D). Each metabolite was identified through specific MS-fragments for *O*-sulfated serotonin (*m/z* = 79.9582, the loss of SO_3_) and the specific fragmentation of *N*-sulfated serotonin (*m/z* = 95.9749, loss of NSO_3_) [32]. Structurally similar metabolites that also contain primary amines can easily be distinguished by analysis of these specific mass spectrometric fragments to identify the correct sulfation site.

### 2.2. Construction of the Sulfate Library

**Figure 3:**
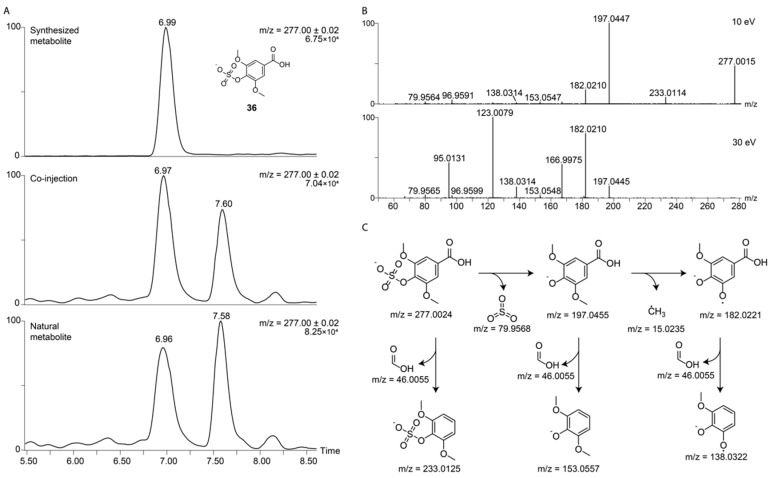
Validation and characterization of sulfated syringic acid (**36**) in a human urine sample. (**A**) Representative co-injection experiments. The top chromatogram represents the EIC of the synthesized compound **36**, the middle chromatogram the EIC of the co-injection experiment of the synthetic and natural molecule **36**, and the bottom chromatogram is EIC for **36**; (**B**) Two fragmentation pattern for syringic acid sulfate (**36**) (10 eV and 30 eV); (**C**) Proposed fragmentation of **36**.

Our developed and validated procedure for preparation of sulfated metabolites was, then, applied for a large set of biologically relevant phenols. We synthesized a series of sulfated compounds of structurally diverse hydroxylated and phenolic compounds to continue building up our already started in-house compound library of 24 sulfated metabolites, which were chemically synthesized in previous studies [19,33]. The selected new substrates were either based on our previous studies, for which we proposed metabolite structures based on MS fragmentation spectra as compared with databases or metabolites that were part of a fecal metabolite library purchased from MetaSci. Using Method B, we prepared 38 new *O*-sulfated metabolites that also contained other functionalities such as carboxylic acids, as well as primary and secondary (Table 1). We focused on metabolites with a single reactive alcohol or phenol. Furthermore, each compound was fragmented at two different voltages (10 eV and 30 eV) to obtain comprehensive mass spectrometric fragmentation spectra, as commonly reported in metabolite databases. This structural information for all prepared sulfated compounds is now available for the scientific community. In addition, we are uploading this information to MS/MS fragmentation spectra databases. We have included the top five fragmentation peaks with the highest intensity from this obtained MS/MS spectra in Table 1, which builds the basis for straightforward compound validation in future studies. To the best of our knowledge, this is the largest chemically synthesized collection of sulfated metabolites that has been reported yet. An example for co-injection experiments of a synthesized metabolite using Method B with the natural compound in urine samples is illustrated for sulfated syringic acid (**36**) in Figure 3. We selected **36** as a representative and realistic example, as this urine sample also contains an additional regioisomer with the same *m*/*z* values but with a different retention time (Figure 3A). The extracted ion chromatogram (EIC) traces in this experiment demonstrate that the synthesized compound and the natural metabolite eluting at 6.99 min are identical due to the same retention time and fragmentation pattern. This realistic example demonstrates the efficient structure validation utilizing our method to distinguish two regioisomers. The chemical structure for the regioisomer at 7.58 min was not determined. The mass spectrometric fragmentation spectra for both applied voltages of **36** fit perfectly to the predicted fragments (Figure 3B,C).

### 2.3. Separation of Regioisomers

**Figure 4:**
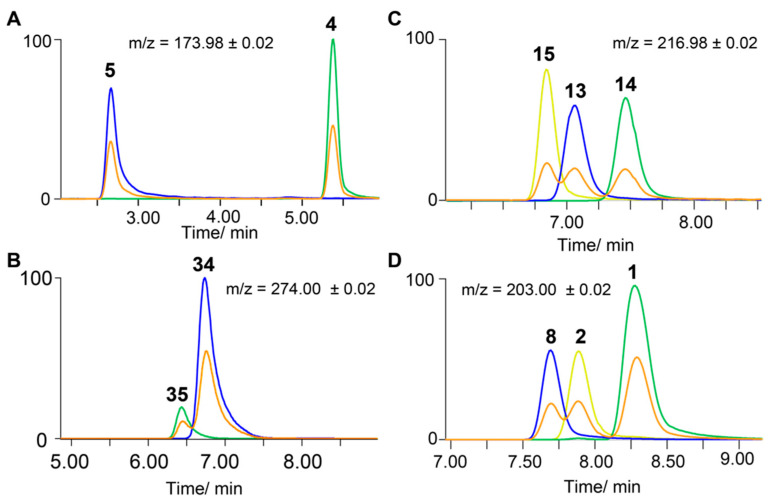
Distinction of representative structural regioisomers prepared with presented strategy Method B. Chromatographic separation of standard mixtures are depicted in orange. (**A**) Extracted ion chromatogram (EIC) traces of the UPLC-MS analysis of 2-hydroxypyridine sulfate (**5**) and 3-hydroxypyridine sulfate (**4**); (**B**) EIC traces of the UPLC-MS analysis of 3-hydroxyhippuric acid sulfate (**34**) and 4-hydroxyhippuric acid sulfate (**35**); (**C**) EIC traces of the UPLC-MS analysis 2-hydroxybenzoic acid sulfate (**14**), 3-hydroxybenoic acid sulfate (**15**), and 4 hydroxybenzoic acid (**16**); (**D**) Injection of 2-methoxyphenol sulfate (**8**), 3-methoxyphenol sulfate (**1**), and 4-methoxyphenol sulfate (**2**).

Another advantage of our method is the ability to efficiently validate and distinguish structural regioisomers. As described above, metabolites with the same chemical formula can generally not be distinguished only based on their MS/MS fragmentation pattern. Chromatographic properties and retention time are difficult to predict for LC-MS-based analysis of similar compounds as a retention index (RI) can currently only be used for gas chromatography coupled to mass spectrometry (GC-MS) analysis [34]. Sulfated phenolic aromatic compounds with the same chemical formula commonly have different substitution pattern such as ferulic acid sulfate and isoferulic acid sulfate which can be present in all human sample types. Despite minor structural differences, these compounds have different bioactive properties, as only ferulic acid sulfate has been described to reduce blood pressure in mice [35]. Depicted in Figure 4 are four different examples of regioisomers that can only be distinguished through their different chromatographic properties. Interestingly, these similar structures can either result in baseline separated or closely eluting and overlapping peaks. Regioisomers 2-hydroxypyridine sulfate (**4**) and 3-hydroxypyridine sulfate (**5**) have a retention time difference of over 2.5 min and can clearly be separated (Figure 4A). This is uncharacteristic as compared with other structural isomers. For example, 3-hydroxyhippuric acid sulfate (**34**) and 4-hydroxyhippuric acid sulfate (**35**) have similar retention times and co-elute (Figure 4B). Single UPLC-MS analyses of each prepared standard demonstrate separation of both peaks. Co-injection experiments at equimolar concentrations can be used to distinguish these metabolites in complex biological matrices. The three metabolites, 2-hydroxybenzoic acid sulfate (**13**), 3-hydroxybenzoic acid sulfate (**14**), and 4-hydroxybenzoic acid sulfate (**15**), also have similar retention times but each structure can clearly be assigned using synthetic reference standards (Figure 4C). A similar example demonstrating the versatility of our method is the facile identification of 2-methoxyphenol sulfate (**8**), 3-methoxyphenol sulfate (**1**), and 4-methoxyphenol sulfate (**2**) using authentic standards (Figure 4D).

### 2.4. Metabolite Library and Significance

Upon the validation of our standard preparation procedure, we have compiled a comprehensive in-house library of 62 authentic sulfated compounds including these 38 additional sulfated metabolites prepared in this study (Supplementary Materials, Table S1). The library contains biochemically relevant compounds including metabolites from human-microbiota co-metabolism or dietary compounds produced by the microbiome. Several of these new metabolites were prepared from a fecal metabolite library of 540 metabolites purchased from MetaSci that included phenolic and bioactive compounds. The colon is the part of the human body with the largest population of bacteria, and therefore metabolite analysis of fecal samples provides the best possibility to study gut microbiota metabolism. Analysis of metabolites in fecal samples will provide more detailed insights in the metabolic interaction of the human host and its gut bacteria. For example, 3-hydroxyhippuric acid and 4-hydroxyhippuric acid have been widely associated with this co-metabolism as bacteria produce the precursor hippuric acid from food compounds [36]. We have recently identified the presence of their sulfated analogues for the first time and they are now available for inclusion in future metabolomics and microbiome analyses [27]. Other metabolites described as part of the benzoic acid biotransformation are three analogues of hydroxybenzoic acid sulfate (**13**, **14**, and **15**) as well as benzyl alcohol sulfate (**6**) [18]. Furthermore, hippuric acid has been described as a marker for uremic diseases, as it is one of the end products of the detoxification of toluene in the human body [37]. Identification and analysis of additional phase I or phase II analogues can provide further insights on the potential of hippuric acid and its analogues as urinary biomarkers for diseases and may lead to identification of unknown bioactive metabolites.

Hydroxypyridines are another compound class that has been associated with bacterial metabolism. Kaiser et al. described specific bacterial reactions for the biosynthesis of 2-hydroxypyridine, 3-hydroxypyridine, and 4-hydroxypyridine [38]. We have previously identified 3-hydroxypyridine sulfate (**5**) in human samples. Raspberry ketone is a compound produced by yeast and its sulfated analogue (**28**) has not yet been identified in human samples [39]. Having the synthetic standard simplifies the validation of this sulfated metabolite in human samples. Raspberry ketone can also be produced through the phenylalanine degradation pathway [40]. We have also synthesized several compounds that are sulfated analogues of products from bacterial degradation of anthocyanins (vanillin, sinapic acid, or syringic acid) [41,42]. Additionally, *N*-methyltyramine is part of the catecholamine metabolism in the human brain, and its sulfated analogue (**18**) has only been reported in human samples once before [43]. Tyramine is a metabolite of great interest as it is produced by bacteria through decarboxylation of tyrosine and one of its analogues has been identified as a biomarker for the parasite onchocerciasis [30]. 3-Hydroxyphenylacetic acid is a product of the bacterial conversion of *m*-tyramine [44]. Mandelic acid is part of the neurotransmitter metabolism and is produced by the degradation of adrenaline and noradrenaline [45]. The sulfated analogue (**20**) can now be identified in human samples. The two synthesized coumarins, umbelliferone and 4-hydroxycoumarin, are part of the *p*-coumaric acid metabolic pathway [46]. Hypoxanthine is a metabolite that is part of the purine degradation pathway in humans, whereas 4-cyanophenol sulfate (**7**) has been widely described as a pesticide-derived metabolite after detoxification metabolism in plants [47].

The comprehensive analysis of these sulfated metabolites of biological importance in both human and bacterial metabolism requires sulfated standard metabolites to determine the correct metabolic pathway and origin. We have also included compounds that have not yet been detected in human samples but are potential metabolic downstream products. Availability of their standards provides the opportunity to (i) determine their absolute chemical structure and (ii) for precise quantification of these metabolites in human samples.

## 3. Materials and Methods

### 3.1. Materials and Equipment

All reagents and solvents were purchased from Sigma-Aldrich or Fischer Scientific and were used without further purification. Most phenolic or hydroxylated compounds used for Method B were part of a metabolite library purchased from MetaSci (Toronto, ON, Canada). HPLC grade solvents were used for HPLC purification and LC-MS grade for UPLC-MS analysis. Solutions were concentrated in vacuo either on a Speedvac Concentrator Plus System (Eppendorf, Hamburg, Germany) or using the Freezone Benchtop 70040 lyophilizer, 4.5 L (Labconco, Kansas City, MO, USA). Chromatographic purification of products was accomplished using preparative reverse phase HPLC on an Agilent HPLC-1100 series system(Agilent, Santa Clara, CA, United States) equipped with a Waters Atlantis T3 preparative column (5 μm, 10 × 100 mm) at a 2.5 mL/min flow rate. All synthesized compounds using Method A were ≥95% pure as determined by NMR. NMR spectra were recorded on an Agilent 400 MHz spectrometer (1H-NMR: 399.97 MHz and 13C NMR: 100.58 MHz). Chemical shifts are reported in parts per million (ppm) on the δ scale from an internal standard. Multiplicities are abbreviated as follows: s = singlet, d = doublet, t = triplet, q = quartet, and m = multiplet. High-resolution mass spectra were acquired on a Waters SYNAPT G2-S High Definition Mass Spectrometry (HDMS) (Waters, Milford, MA, USA) using an electrospray ionization (ESI) source with a Waters AQCUITY UPLC I class system(Waters, Milford, MA, USA) and equipped with a Waters Acquity UPLC^®^ HSS T3 column (1.8 μm, 100 × 2.1 mm, (Waters, Milford, MA, USA)).

### 3.2. Human Samples

Healthy donor urine samples were obtained in accordance with the World Medical Association Declaration of Helsinki and all healthy donors gave written informed consent (Ethical approval number: Dnr 2017/290-31). All samples were stored at −80 °C.

### 3.3. Chemical Synthesis: Method A

The general procedure is as follows: To a solution of the corresponding hydroxyl compound and 3.0 eq NaOH, 4.0 eq NaHCO_3_ and 2.5 eq SO_3_·NMe_3_ complex were added, as illustrated in Scheme S1. The reaction mixture was stirred at room temperature for 24 h and concentrated using a lyophilizer. The dry crude mixture was purified by preparative HPLC to yield the desired product (details are described in Supplementary Materials, Scheme S1).

### 3.4. Preparation of Sulfates: Method B

Sulfates were prepared by mixing at least 0.2 mg of a hydroxyl molecule with 1.0 eq of NaOH, 3 eq of NaHCO_3_, and 3 eq of SO_3_·NMe_3_. The reaction was stirred for 16 h in an inert gas atmosphere. The solvent of the reaction mixture was removed using a lyophilizer and the remaining solid was re-dissolved in a 5% acetonitrile solution in water and analyzed using UPLC-MS. This method was used for all compounds included in Table 1.

### 3.5. UPLC-MS/MS Analysis

Mass spectrometric analysis was performed on an Acquity UPLC system connected to a Synapt G2 Q-TOF mass spectrometer, both from Waters Corporation (Milford, MA, USA). Data acquisition and analysis was performed using the MassLynx software package v 4.1 (Waters, Milford, MA, USA). The separation was performed on an Acquity UPLC^®^ HSS T3 column (1.8 μm, 100 × 2.1 mm) from Waters Corporation. The mobile phase consisted of the following: (A) 0.1% formic acid in MilliQ water and (B) 0.1% formic acid in LC-MS-grade methanol. The column temperature was 40 °C and the gradient applied was as follows: 0–2 min, 0% B; 2–15 min, 0–100% B; 15–16 min, 100% B; 16–17 min, 100–0% B; 17–21 min, 0%, with a flow rate of 0.2 mL/min.

The samples were introduced into the q-TOF using negative electrospray ionization. The capillary voltage was set to −2.50 kV and the cone voltage was 40 V. The source temperature was 100 °C, the cone gas flow was 50 L/min, and the desolvation gas flow was 600 L/h. The instrument was operated in MSE mode, the scan range was *m/z* = 50–1200, and the scan time was 0.3 s. In low energy mode, the collision energy was 10 eV and in high energy mode the collision energy was ramped between 25 and 45 eV. A solution of sodium formate (0.5 mM in 2-propanol/water, 90:10, *v*/*v*) was used to calibrate the instrument and a solution of leucine-encephalin (2 ng/μL in acetonitrile/0.1% formic acid in water, 50:50, *v*/*v*) was used for the lock mass correction, at an injection rate of 30 s.

For the MS/MS fragmentation analysis, the 10 eV used was a combination of 5 eV on the trap and 5 eV on the transfer in the collision cell, and the 30 eV used was a combination of 10 eV on the trap and 20 eV on the transfer in the collision cell.

## 4. Conclusions

In this study, we present the chemical synthesis of a large library of sulfated metabolites relevant for mass spectrometric structure validation in human samples. By developing a straightforward and high-throughput synthetic procedure, we have synthesized and characterized 38 new sulfated compounds of diverse metabolic scaffolds. This metabolite library can easily be extended by any commercially available or synthetic monohydroxylated and monophenolic compound using Method B. As this compound class has been identified as a signature for microbiota-host co-metabolism, this unique metabolite library can serve to elucidate unknown metabolic interactions. Uncovering new metabolic interaction and further insights into this interspecies metabolism has high potential for identifying unknown bioactive compounds to understand human physiology and disease development linked to microbiota dysbiosis. This sulfated metabolite library is now available for the scientific community for inclusion in standard metabolomics studies.

## Figures and Tables

**Table 1:** Overview of the standard library including 38 metabolites. Description of the chemical formula, *m/z* ratio (in negative mode of ionization), detected retention time, and the top 5 fragments (bigger than 4%) for two different voltages (10 eV and 30 eV). All numbered chemical structures are depicted in Supplementary Materials, Figure S1.

Metabolite	Chemical Formula	*m/z*	Retention Time/min	Fragmentation *m/z* (% of Maximum Intensity)
Phenol sulfate (**3**)	C_6_H_5_O_4_S^−^	172.9914	7.38	10 eV—172.9893 (100), 93.0324 (38), 79.9551 (4) 30 eV—93.0337 (100), 79.9563 (6)
2-Hydroxypyridine sulfate (**4**)	C_5_H_4_NO_4_S^−^	173.9867	5.15	10 eV—173.9857 (100), 94.0289 (89) 30 eV—94.0288 (100)
3-Hydroxypyridine sulfate (**5**)	C_5_H_4_NO_4_S^−^	173.9867	2.53	10 eV—173.9860 (100), 94.0289 (53) 30 eV—94.0288 (100)
Benzyl alcohol sulfate (**6**)	C_7_H_7_O_4_S^−^	187.0071	8.21	10 eV—187.0061 (100), 95.9514 (6) 30 eV - 95.9511 (100), 187.0056 (11), 79.9562 (10), 80.9639 (8), 77.0384 (7)
4-Cyanophenol sulfate (**7**)	C_7_H_4_NO_4_S^-^	197.9867	7.54	10 eV—118.0290 (100), 197.9854 (55) 30 eV—118.0287 (100), 90.0340 (4)
2-Methoxyphenol sulfate (**8**)	C_7_H_7_O_5_S^−^	203.0020	7.67	10 eV—203.0009 (100), 123.0445 (30), 108.0207 (6), 79.9561 (5) 30 eV—108.0205 (100), 123.0442 (15), 79.9562 (9)
3-Methoxyphenol sulfate (**1**)	C_7_H_7_O_5_S^−^	203.0020	8.51	10 eV—203.0015 (100), 123.0444 (28) 30 eV—108.0213 (100), 123.0444 (59), 79.9564 (5)
4-Methoxyphenol sulfate (**2**)	C_7_H_7_O_5_S^−^	203.0020	8.15	10 eV—203.0011 (100), 187.9773 (13), 123.0443 (8), 108.0208 (5), 79.9563 (5) 30 eV—108.0212 (100), 79.9564 (19), 123.0442 (4)
3-Hydroxy-2-methyl-4-pyrone sulfate (**9**)	C_6_H_5_O_6_S^−^	204.9812	5.30	10 eV—204.9790 (100), 125.0223 (50), 80.9630 (4) 30 eV—125.0236 (100), 97.0285 (62), 79.9561 (38), 69.0330 (13), 80.9640 (12)
4,5-Dimethyl-3-hydroxy-2,5-dihydrofuran-2-one sulfate (**10**)	C_6_H_7_O_6_S^−^	206.9969	7.28	10 eV—206.9946 (100), 127.0381 (24), 135.0099 (8) 30 eV—127.0391 (100), 135.0110 (36), 79.9562 (36), 80.9641 (28), 99.0443 (21)
2-Hydroxyacetophenone sulfate (**11**)	C_8_H_7_O_5_S^−^	215.0020	8.28	10 eV—215.0006 (100), 135.0441 (99) 30 eV—135.0438 (100), 93.0331 (44)
Hypoxanthine sulfate (**12**)	C_5_H_3_N_4_O_4_S^−^	215.9953	2.58	10 eV—136.0325 (100), 215.9891 (99), 135.0298 (13) 30 eV—136.0331 (100), 93.0268 (41), 92.0240 (16), 135.0310 (13), 109.0228 (4)
2-Hydroxybenzoic acid sulfate (**13**)	C_7_H_5_O_6_S^−^	216.9812	7.00	10 eV—137.0223 (100), 93.0325 (15), 216.9792 (15);
3-Hydroxybenzoic acid sulfate (**14**)	C_7_H_5_O_6_S^−^	216.9812	7.19	10 eV—216.9789 (100), 137.0223 (49), 93.0326 (5) 30 eV—93.0337 (100), 137.0232 (44)
4-Hydroxybenzoic acid sulfate (**15**)	C_7_H_5_O_6_S^−^	216.9812	6.80	10 eV—216.9801 (100), 137.0225 (87), 93.0336 (12), 172.9905 (8), 96.9591 (5) 30 eV—93.0337 (100), 137.0235 (27)
4-Hydroxyquinoline sulfate (**16**)	C_9_H_9_NO_4_S^−^	224.0023	8.09	10 eV—224.000 (100), 144.0433 (99) 30 eV—144.0447 (100)
4-Acetamidophenol sulfate (**17**)	C_8_H_8_NO_5_S^−^	230.0129	5.65	10 eV—230.0120 (100), 150.0556 (42) 30 eV—150.0552 (100), 107.0368 (53), 108.0448 (5)
*N*-Methyltyramine sulfate (**18**)	C_9_H_12_NO_4_S^−^	230.0493	7.56	10 eV—230.0467 (100), 109.9893 (8) 30 eV—79.9561 (100), 109.9906 (21), 150.0916 (8), 80.9639 (5)
4-Hydroxyphenylacetic acid sulfate (**19**)	C_8_H_7_O_6_S^−^	230.9969	7.09	10 eV—230.9966 (100), 151.0398 (31) 30 eV—107.0497 (100),151.0393 (48), 105.0337 (7), 79.9564 (6)
Mandelic acid sulfate (**20**)	C_8_H_7_O_6_S^−^	230.9969	7.33	10 eV—230.9959 (100), 96.9591 (75), 151.0391 (34) 30 eV—96.9590 (100), 151.0390 (13), 107.0492 (8)
3-Hydroxyphenylacetic acid sulfate (**21**)	C_8_H_7_O_6_S^−^	230.9969	7.18	10 eV—230.9948 (100), 187.0047 (70), 107.0481 (22), 79.9553 (5) 30 eV—107.0492 (100), 79.9564 (16)
Vanillin sulfate (**22**)	C_8_H_7_O_6_S^−^	230.9969	7.42	10 eV—151.0392 (100), 230.9958 (44), 136.0157 (23) 30 eV—136.0157 (100), 92.0259 (15). 151.0391 (13), 108.0206 (12)
5-Aminosalicylic acid sulfate (**23**)	C_7_H_6_NO_6_S^−^	231.9921	5.98	10 eV—231.9896 (100), 152.0331 (85), 150.0174 (57), 213.9791 (49), 106.0280 (17) 30 eV—79.9565 (100), 106.0289 (48), 78.0341 (40), 80.9642 (38), 108.0447 (35)
4-Hydroxycoumarin sulfate (**24**)	C_6_H_5_O_6_S^−^	240.9812	9.36	10 eV—161.0228 (100), 204.9792 (15), 117.0323 (6) 30 eV—117.0338 (100), 161.0236 (55)
Umbelliferone sulfate (**25**)	C_6_H_5_O_6_S^−^	240.9812	7.89	10 eV—161.0226 (100), 240.9791 (40) 30 eV—161.0237 (100), 133.0286 (21), 105.0337 (7), 77.0385 (5), 89.0385 (4)
trans-3-Hydroxycinnamic acid sulfate (**26**)	C_6_H_7_O_6_S^−^	242.9969	8.48	10 eV—242.9962 (100), 163.0393 (54) 30 eV—119.0497 (100), 163.0395 (67)
trans-4-Hydroxycinnamic acid sulfate (**27**)	C_6_H_7_O_6_S^−^	242.9969	7.99	10 eV—163.0379 (100), 242.9947 (64), 119.0481 (16) 30 eV—119.0495 (100), 163.0393 (10)
Raspberry ketone sulfate (**28**)	C_10_H_11_O_5_S^−^	243.0333	8.34	10 eV—243.0327 (100), 163.0750 (13) 30 eV—163.0755 (100), 57.0329 (81), 79.9561 (45), 80.9639 (4)
D-3-Phenyllactic acid sulfate (**29**)	C_9_H_9_O_6_S^−^	245.0125	10.20	10 eV—245.011 (100), 96.9578 (59), 165.0534 (21), 244.9520 (6) 30 eV—96.9591 (100), 147.0442 (43), 103.0542 (20), 165.0549 (16), 128.9950 (7)
3-(3-Hydroxyphenyl)propionic acid sulfate (**30**)	C_9_H_9_O_6_S^−^	245.0125	8.08	10 eV—245.0101 (100), 96.9578 (58), 165.0534 (20) 30 eV—96.9591 (100), 147.0443 (42), 103.0542 (19), 165.0552 (18), 119.0493 (15)
5-Methoxysalicylic acid sulfate (**31**)	C_8_H_7_O_7_S^−^	246.9918	7.58	10 eV—167.0325 (100), 152.0090 (16), 246.9891 (15), 108.0195 (11) 30 eV—108.0207 (100), 152.0104 (27), 167.0341 (7)
Serotonin sulfate (**32**)	C_10_H_11_N_2_O_4_S^−^	255.0445	5.92	10 eV—255.0428 (100), 175.0871 (7), 254.9162 (6), 219.8448 (5)
4-Hydroxy-3-methoxyphenylacetic acid sulfate (**33**)	C_9_H_9_O_7_S^−^	261.0074	7.46	10 eV—261.0052 (100), 181.0484 (32), 217.0153 (9) 30 eV—137.0602 (100), 122.0366 (80), 181.0496 (23), 79.9563 (10), 105.0337 (6)
3-Hydroxyhippuric acid sulfate (**34**)	C_9_H_8_NO_7_S^−^	274.0027	6.73	10 eV—274.0007 (100), 194.0435 (17), 150.0539 (7) 30 eV—150.0551 (100), 93.0335 (38), 194.0448 (16)
4-Hydroxyhippuric acid sulfate (**35**)	C_9_H_8_NO_7_S^−^	274.0027	6.43	10 eV—274.0005 (100), 194.0435 (68), 100.0017 (9) 30 eV—93.0335 (100), 100.0029 (97), 74.0233 (39), 150.0550 (19), 194.0448 (10)
Syringic acid sulfate (**36**)	C_9_H_9_O_8_S^−^	277.0024	6.99	10 eV—197.0447 (100), 277.0015 (47), 182.0210 (17), 233.0114 (8), 96.9591 (5) 30 eV—123.0079 (100), 182.0211 (79), 95.0131 (43), 166.9975 (41), 197.0445 (14)
Sinapic acid sulfate (**37**)	C_11_H_11_O_8_S^−^	303.0180	8.11	10 eV—223.0601 (100), 303.0169 (39), 208.0366 (12), 259.0269 (5) 30 eV—193.0134 (100), 121.0287 (71), 149.0236 (64), 208.0368 (61), 164.0471 (51)
3-Hydroxyflavone sulfate (**38**)	C_15_H_9_O_6_S^−^	317.0125	12.8	10 eV—237.0574 (100), 317.0130 (58) 30 eV—237.0554 (100), 181.0655 (83), 180.0574 (71), 209.060 (39), 153.0701 (17)

## Supplementary Materials

The following supplementary materials are available online at https://www.mdpi.com/2218-1989/10/10/415/s1, Figure S1: Chemical structures of the molecules synthesized, Table S1: Names of all sulfated metabolites. Scheme S1: Chemical synthesis (Method A).
