# Supplementary material for: Rapid Preparation of a Large Sulfated Metabolite Library for Structure Validation in Human Samples
Source: Metabolites. 2020 Oct 16;10(10):415. doi: 10.3390/metabo10100415 (PMC7603051; doi:10.3390/metabo10100415)
Supplement: Supplementary file 1 [file metabolites-10-00415-s001.pdf]

Supplementary materials

Rapid preparation of a large sulfated metabolite library for structure validation in human samples

Mario S.P. Correia [1], Weifeng Lin [1], Arash J. Aria [1], Abhishek Jain [1] and Daniel Globisch[1,*]

[1] Dept. Medicinal Chemistry, Science for Life Laboratory, Uppsala University, Box 574, SE-75123 Uppsala, Sweden

* Correspondence: Daniel.globisch@scilifelab.uu.se; Tel.: +46 18-471-4287

Contents

Supplementary figures……………………………………………………………………….…2

Supplementary tables…………………………………………………………………….......3

Supplementary schemes……………………………………………………………………..4

Chemical synthesis………………………………………………………………...............5

NMR spectra…………………………………………………………………………………..6

Supplementary figures

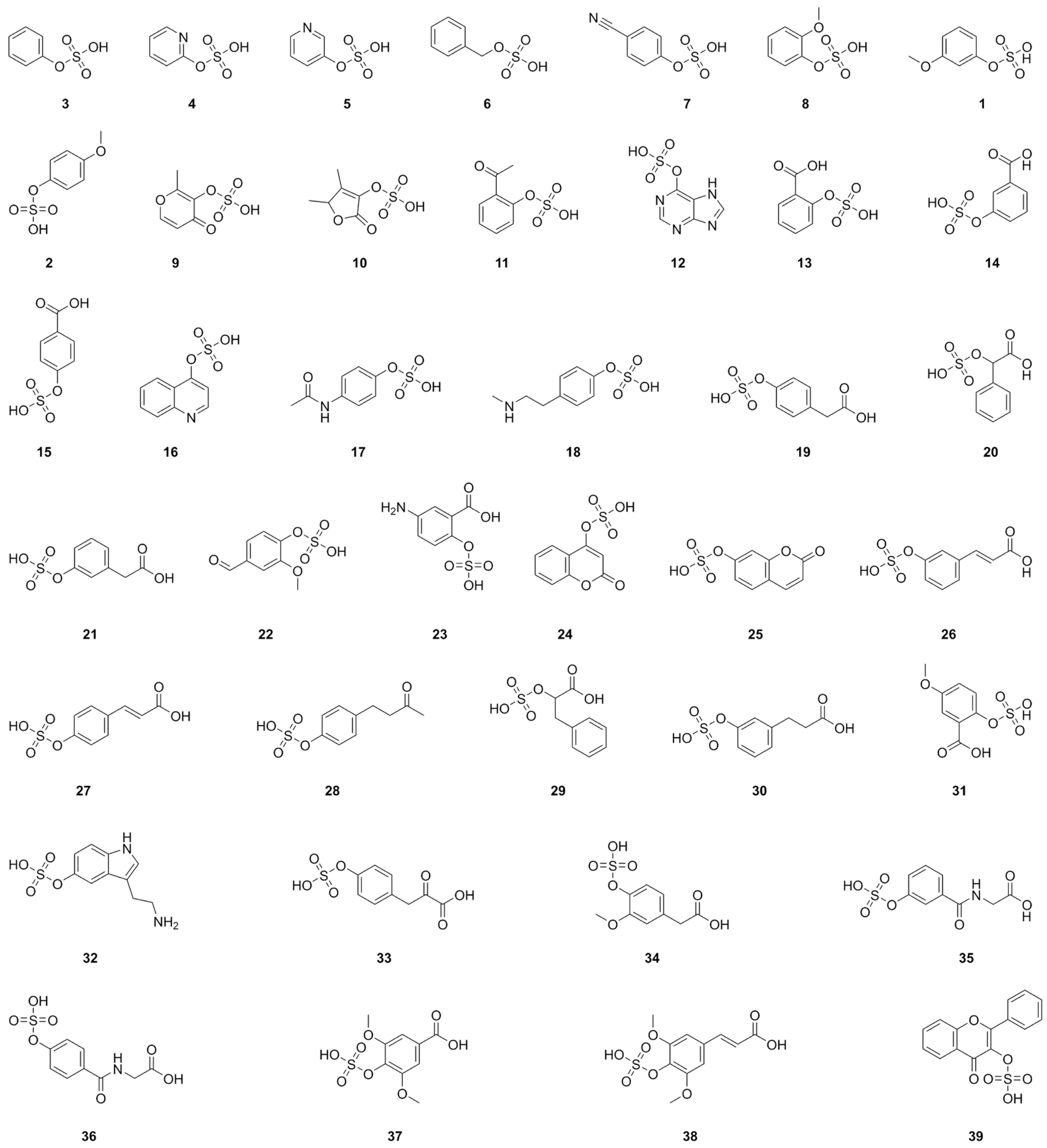

Figure S1 – Overview of the structures of the molecules synthesized. Molecules organized by m/z ratio.

Supplementary tables

Table S1 – All sulfated metabolites

**Newly synthesized sulfated metabolites**	**In-house sulfated metabolites**
Phenol sulfate (**3**)	p-Cresol sulfate
2-Hydroxypyridine sulfate (**4**)	Ferulic acid sulfate
3-Hydroxypyridine sulfate (**5**)	Mannose-6-sulfate
Benzyl alcohol sulfate (**6**)	4-Hydroxy-3-methoxyphenylglycol sulfate
4-Cyanophenol sulfate (**7**)	4-Nitrophenol sulfate
2-Methoxyphenol sulfate (**8**)	N-Acetylserotonin sulfate
3-Methoxyphenol sulfate (**1**)	Tyrosine sulfate
4-Methoxyphenol sulfate (**2**)	Resorcinol sulfate
3-Hydroxy-2-methyl-4-pyrone sulfate (**9**)	Vanillic acid sulfate
4,5-Dimethyl-3-hydroxy-2,5-dihydrofuran-2-one sulfate (**10**)	N-Acetyltyramine sulfate
2-Hydroxyacetophenone sulfate (**11**)	Isovanillic acid sulfate
Hypoxanthine sulfate (**12**)	Indoxyl sulfate
2-Hydroxybenzoic acid sulfate (**13**)	p-Coumaric acid sulfate
3-Hydroxybenzoic acid sulfate (**14**)	4-Methylumbelliferyl sulfate
4-Hydroxybenzoic acid sulfate (**15**)	4-Ethylphenyl sulfate
4-Hydroxyquinoline sulfate (**16**)	Methylurolithin sulfate
4-Acetamidophenol sulfate (**17**)	3-Methylindole-5-O-sulfate
N-Methyltyramine sulfate (**18**)	Dopamine-3-sulfate
4-Hydroxyphenylacetic acid sulfate (**19**)	Dopamine-4-sulfate
Mandelic acid sulfate (**20**)	2-Methoxyphenol-4-vinylphenyl sulfate
3-Hydroxyphenylacetic acid sulfate (**21**)	Estrone-3-sulfate
Vanillin sulfate (**22**)	Dehydroisoandroesterone-3-sulfate
5-Aminosalicylic acid sulfate (**23**)	Cholesterol sulfate
4-Hydroxycoumarin sulfate (**24**)	4-Nitrocatechol sulfate
Umbelliferone sulfate (**25**)	
trans-3-Hydroxycinnamic acid sulfate (**26**)	
trans-4-Hydroxycinnamic acid sulfate (**27**)	
Raspberry ketone sulfate (**28**)	
D-3-Phenyllactic acid sulfate (**29**)	
3-(3-Hydroxyphenyl)propionic acid sulfate (**30**)	
5-Methoxysalicylic acid sulfate (**31**)	
Serotonin sulfate (**32**)	
4-Hydroxy-3-methoxyphenylacetic acid sulfate (**33**)	
3-Hydroxyhippuric acid sulfate (**34**)	
4-Hydroxyhippuric acid sulfate (**35**)	
Syringic acid sulfate (**36**)	
Sinapic acid sulfate (**37**)	
3-Hydroxyflavone sulfate (**38**)	

Supplementary schemes

Scheme S1 – Chemical synthesis reaction of 3-methoxyphenol-*O*-sulfate (1) and 4-methoxyphenol-*O*-sulfate

Synthesis of 3-Methoxyphenol-*O*-sulfate (1)

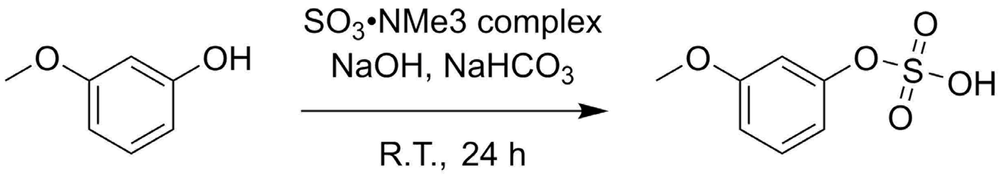

Synthesis of 4-Methoxyphenol-*O*-sulfate (2)

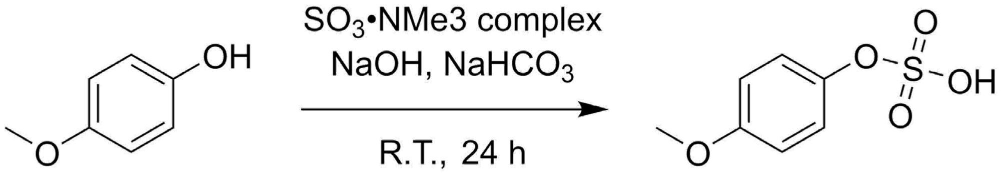

Chemical synthesis

Synthesis of 3-Methoxyphenol-*O*-sulfate

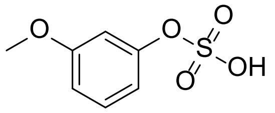

3-methoxyphenol-*O*-sulfate

To a solution of 3-Methoxyphenol (44.2 ul, 0.403 mmol, 1.0 eq) and NaOH (1.2 mL, 1.2 mmol, 3.0 eq), $NaHCO_3$ (135.5 mg, 1.6 mmol, 4.0 eq) and SO_3•NMe_3 complex (140 mg, 1.0 mmol, 2.5 eq) were added, as illustrated in Scheme 1. The reaction mixture was stirred (800 rpm) at room temperature for 24 hours and concentrated in a freeze drier overnight (-51 °C, 0.05 mbar). The crude product was subjected to HPLC purification [r.t. = 22.5 min, 0-5 min (0% B), 5-20 min (0-100 %B), 20-30 min (0% B) at a flow of 2.5 mL/min; buffer A = ammonium acetate 5 mM (water) and buffer B = ammonium acetate 5 mM (MeOH)] to afford 3-Methoxyphenol-O-sulfate.

^{1}H NMR (400 MHz, D_2O) δ (ppm) = 3.83 (3H, s), 6.53-6.65 (3H, m) 7.25 (1H, t, *J* = 8.2 Hz);

^{13}C NMR (151 MHz, D_2O) δ (ppm) = 55.28, 101.42, 106.12, 108.24, 130.62, 156.75, 160.18;

HRMS (ESI-) calculated for $C_7H_7O_5S^-$ $(M-H)^-$: 203.0014, found: 203.0057.

Synthesis of 4-Methoxyphenol-*O*-sulfate

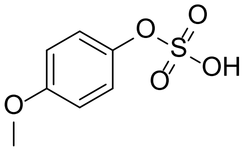

4-methoxyphenol-*O*-sulfate

To a solution of 4-Methoxyphenol (50 mg, 0.403 mmol, 1.0 eq) and NaOH (1.2 mL, 1.2 mmol, 3.0 eq), $NaHCO_3$ (135.5 mg, 1.6 mmol, 4.0 eq) and SO_3•NMe_3 complex (140 mg, 1.0 mmol, 2.5 eq) were added, as illustrated in Scheme 1. The reaction mixture was stirred (800 rpm) at room temperature for 24 hours and concentrated in a freeze drier overnight (-51 °C, 0.05 mbar). The crude product was subjected to HPLC purification [r.t. = 22.0 min, 0-5 min (0% B), 5-20 min (0-100 %B), 20-30 min (0% B) at a flow of 2.5 mL/min; buffer A = ammonium acetate 5 mM (water) and buffer B = ammonium acetate 5 mM (MeOH)] to afford 4-Methoxyphenol-O-sulfate.

^{1}H NMR (400 MHz, D_2O) δ (ppm) = 3.70 (3H, s), 6.80 (2H, d, *J* = 9.0 Hz), 7.14 (2H, d, *J* = 9.0 Hz);

^{13}C NMR (151 MHz, D_2O) δ (ppm) = 54.57, 113.66, 122.38, 146.05, 156.98;

HRMS (ESI-) calculated for $C_7H_7O_5S^-$ $(M-H)^-$: 203.0014, found: 203.0047.

NMR spectra

3-Methoxyphenol sulfate

^{1}H NMR, ^{13}C NMR, COSY, HSQC, HSBC

4-Methoxyphenol sulfate

^{1}H NMR, ^{13}C NMR, COSY, HSQC, HSBC

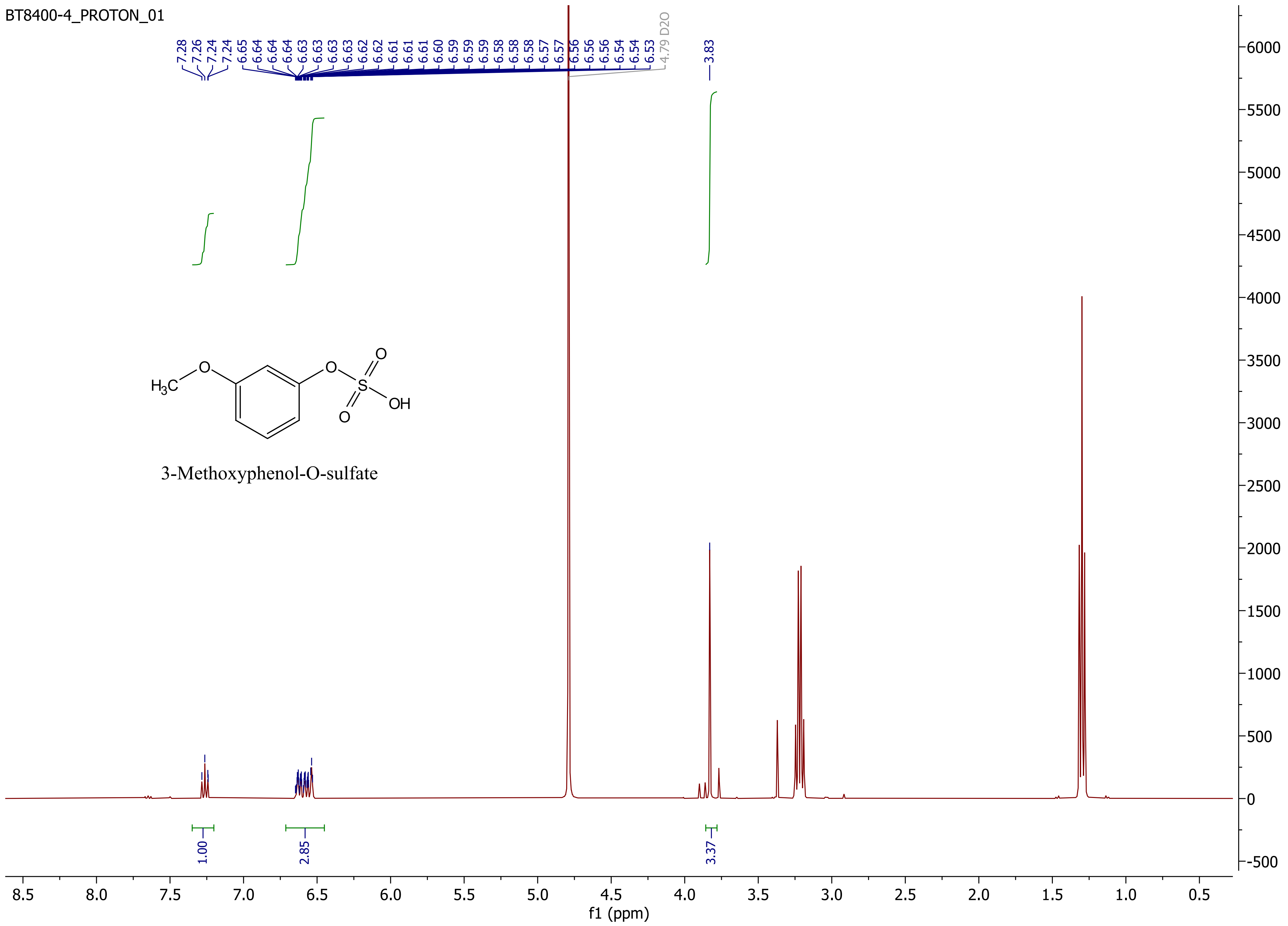
BT8400-4_PROTON_01
7.28 7.26 7.24 7.24 6.65 6.64 6.64 6.64 6.63 6.63 6.63 6.63 6.62 6.62 6.61 6.61 6.61 6.60 6.59 6.59 6.59 6.58 6.58 6.58 6.57 6.57 6.56 6.56 6.56 6.54 6.54 6.53
4.79 D2O
3.83
H3C O O S O O OH
3-Methoxyphenol-O-sulfate
1.00
2.85
3.37
f1 (ppm)

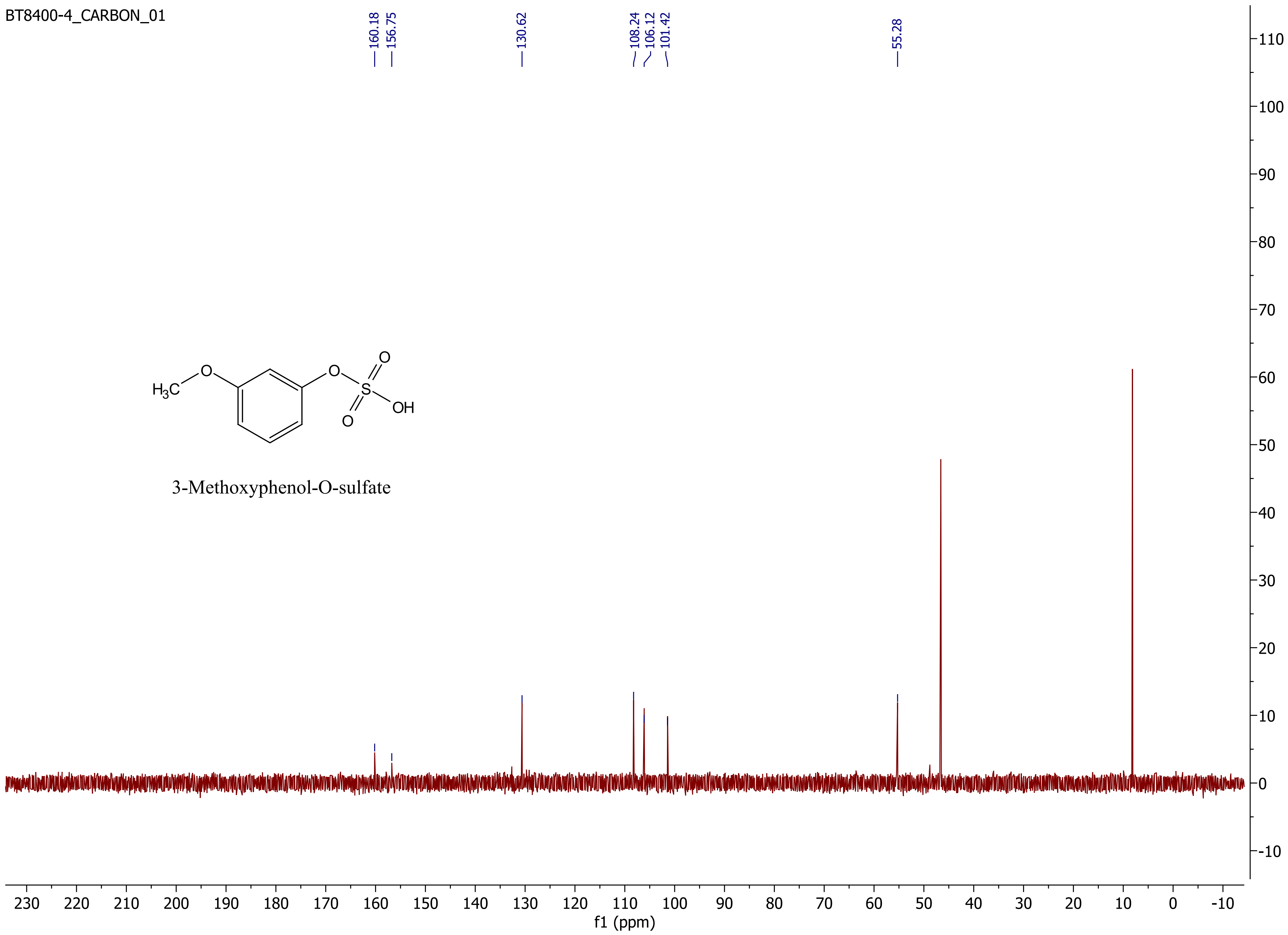

3-Methoxyphenol-O-sulfate

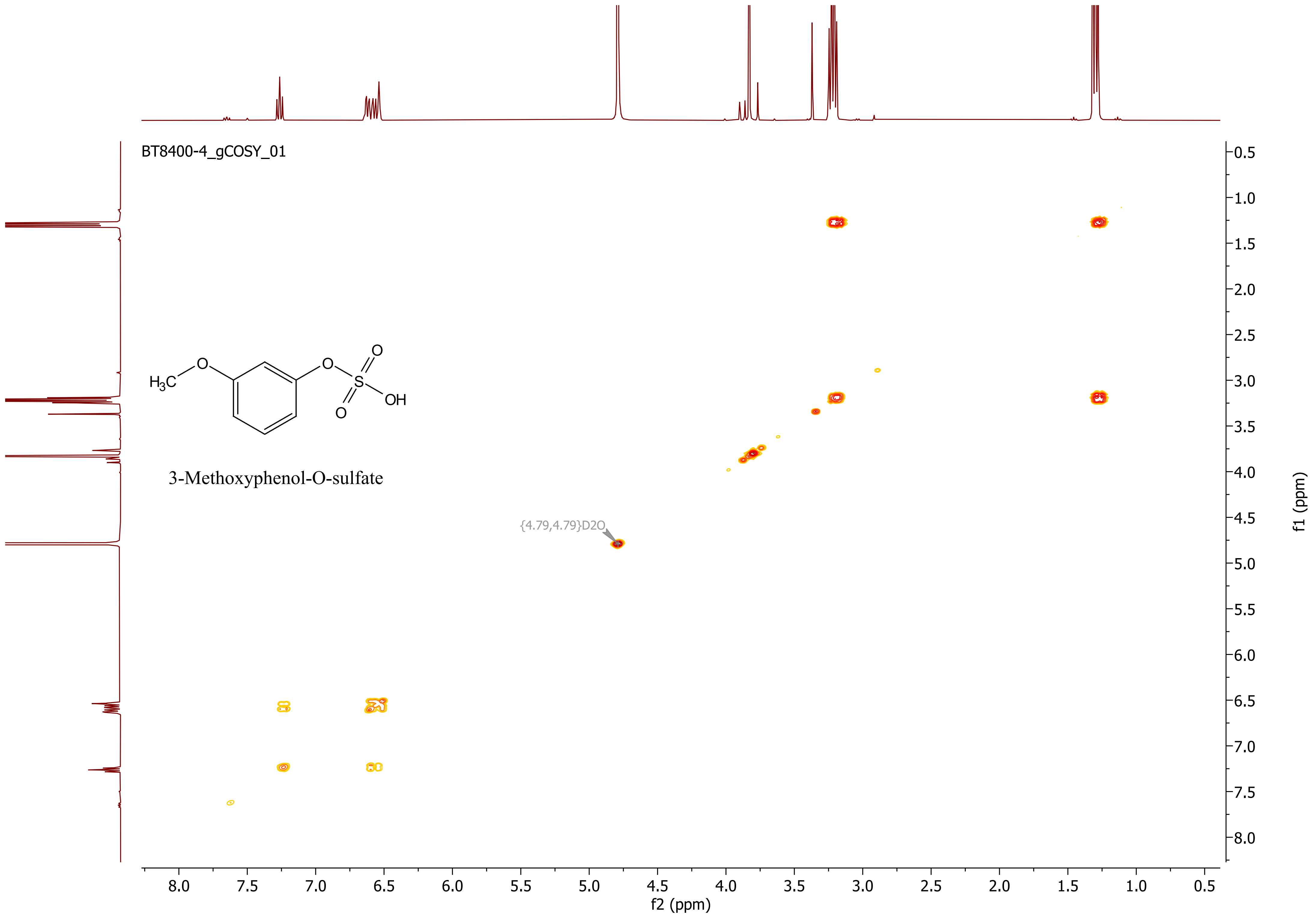
BT8400-4_gCOSY_01
H3C
O
O
S
O
OH
O
3-Methoxyphenol-O-sulfate
{4.79,4.79}D2O
8.0
7.5
7.0
6.5
6.0
5.5
5.0
4.5
4.0
3.5
3.0
2.5
2.0
1.5
1.0
0.5
f2 (ppm)
f1 (ppm)

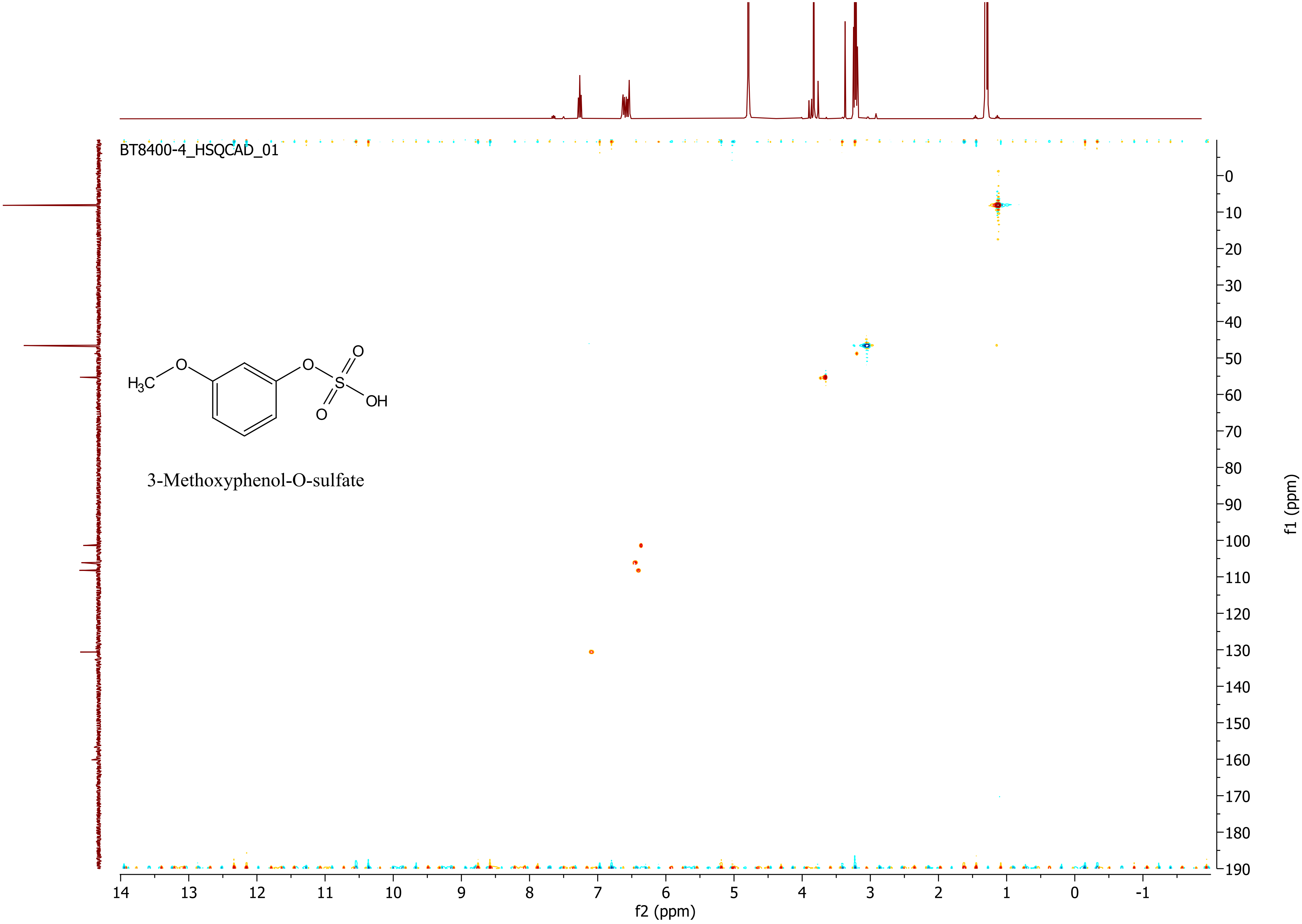
BT8400-4_HSQCAD_01
H3C
O
O
S
O
O
OH
3-Methoxyphenol-O-sulfate
f2 (ppm)
f1 (ppm)

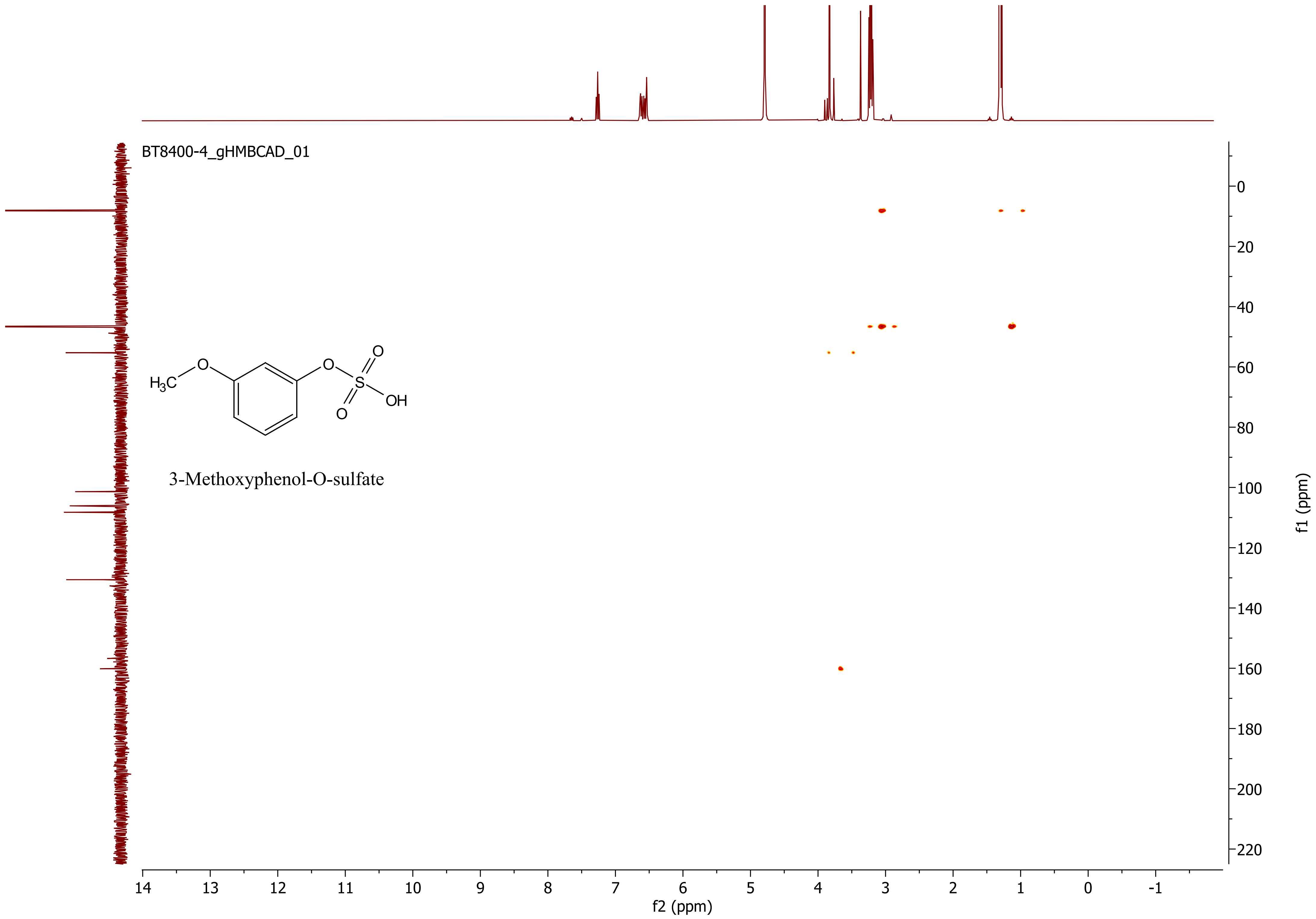
BT8400-4_gHMBCAD_01
3-Methoxyphenol-O-sulfate
H3C
O
S
OH
f1 (ppm)
f2 (ppm)
14 13 12 11 10 9 8 7 6 5 4 3 2 1 0 -1
0 20 40 60 80 100 120 140 160 180 200 220

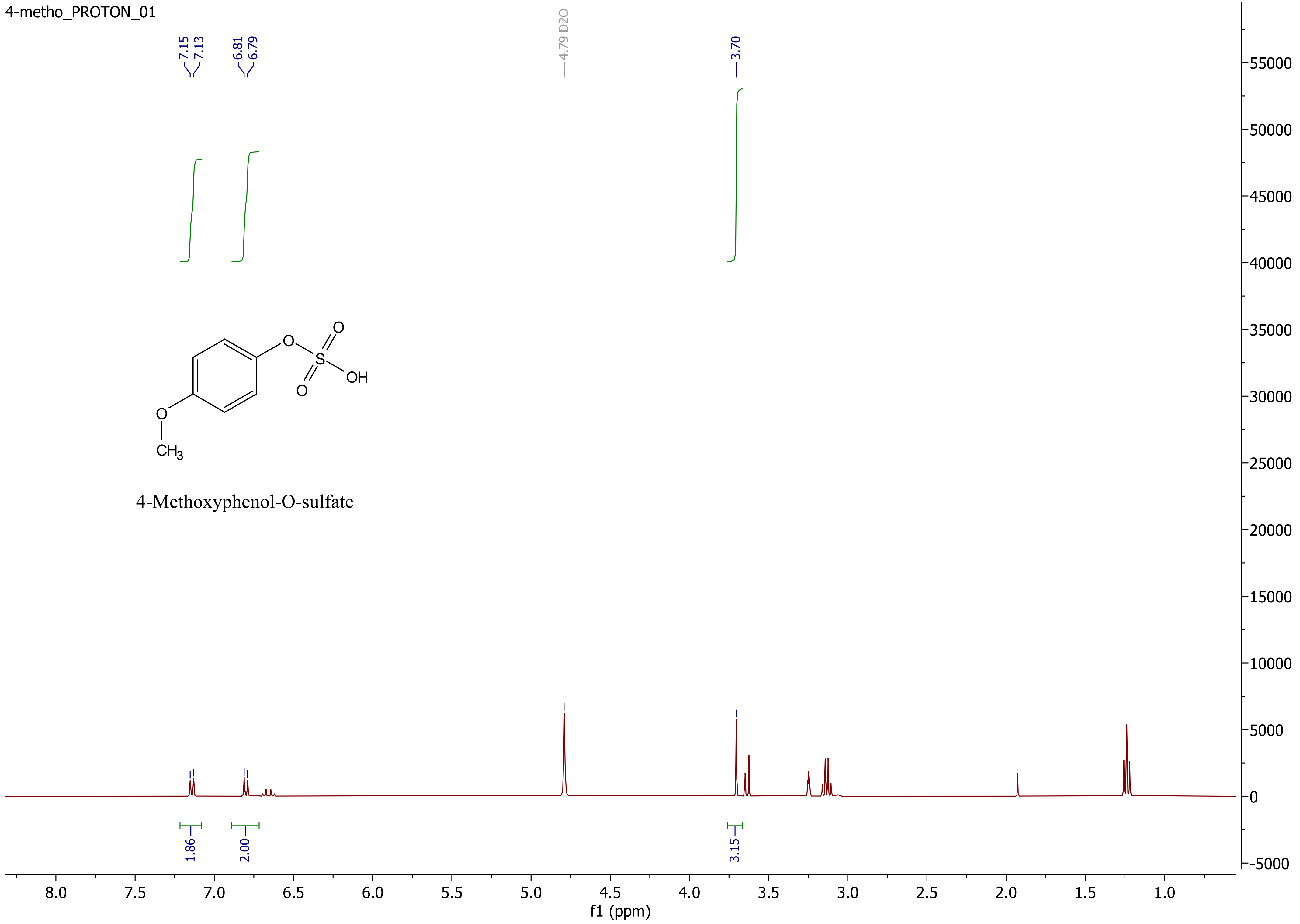
4-metho_PROTON_01
7.15
7.13
6.81
6.79
4.79 D2O
3.70
O
S
OH
O
O
O
CH3
4-Methoxyphenol-O-sulfate
1.86
2.00
3.15
55000
50000
45000
40000
35000
30000
25000
20000
15000
10000
5000
0
-5000
8.0
7.5
7.0
6.5
6.0
5.5
5.0
4.5
4.0
3.5
3.0
2.5
2.0
1.5
1.0
f1 (ppm)

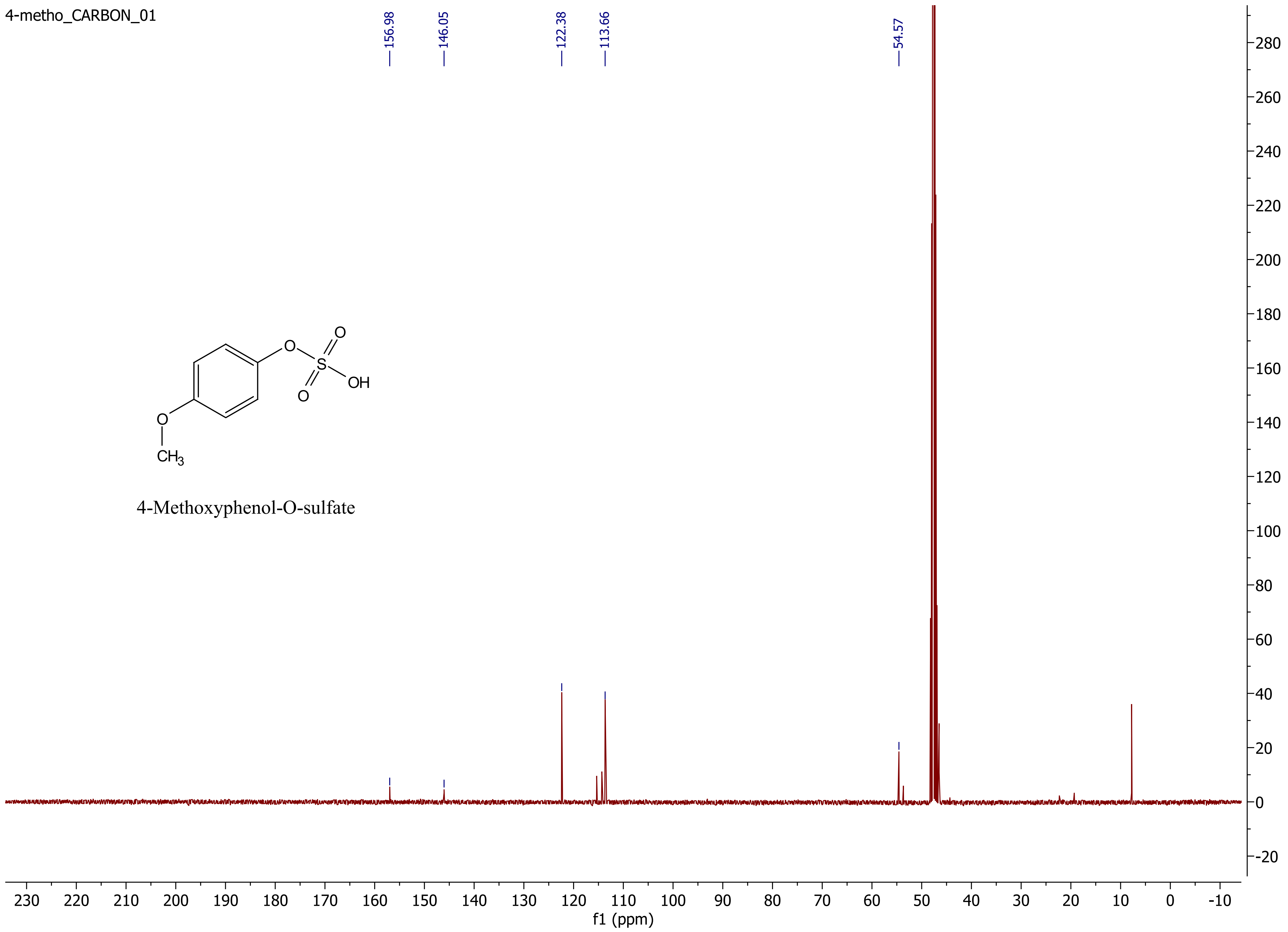
4-metho_CARBON_01
156.98
146.05
122.38
113.66
54.57
4-Methoxyphenol-O-sulfate
f1 (ppm)

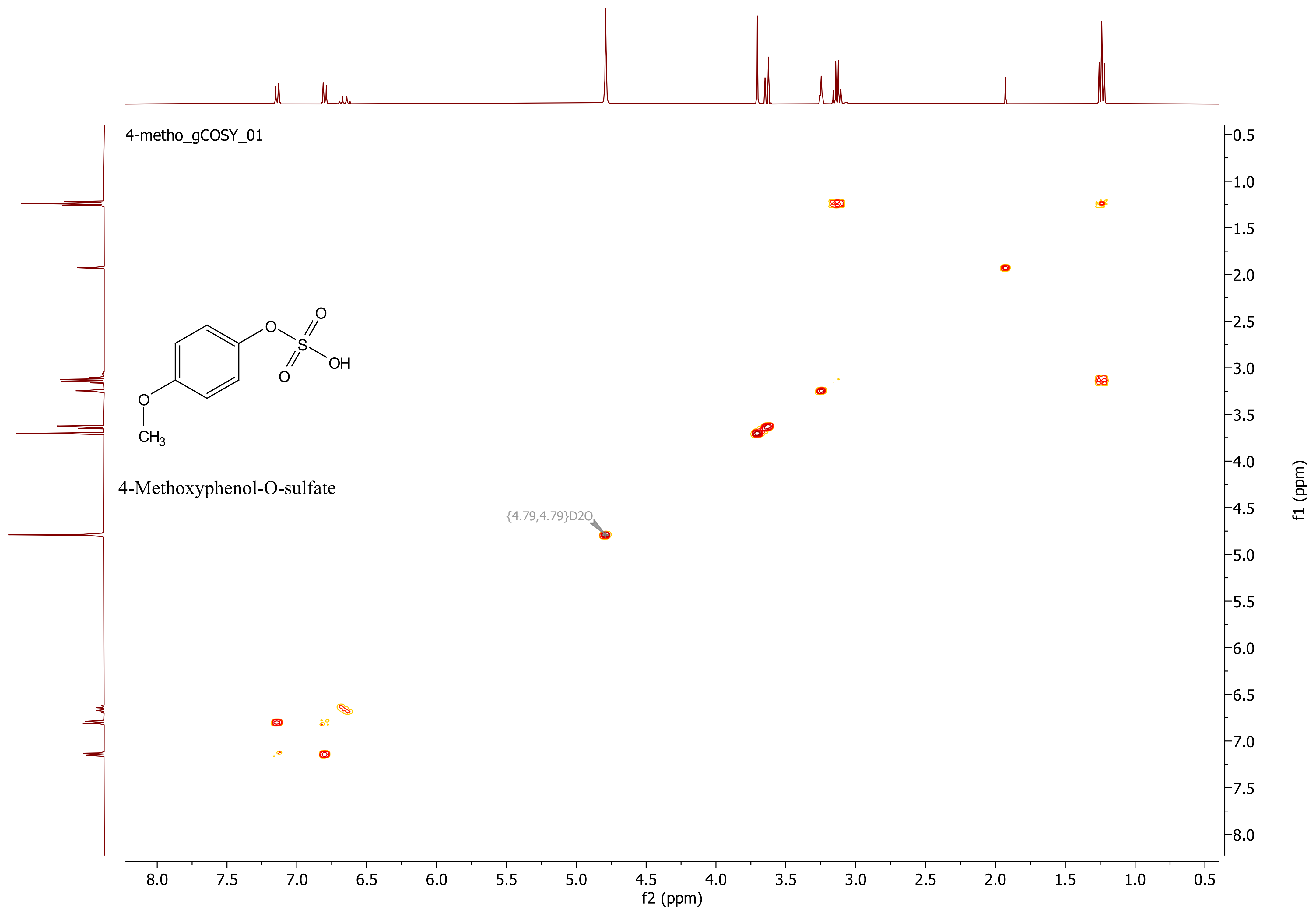
4-metho_gCOSY_01
O
S
OH
O
O
O
CH3
4-Methoxyphenol-O-sulfate
{4.79,4.79}D2O
f2 (ppm)
f1 (ppm)
8.0
7.5
7.0
6.5
6.0
5.5
5.0
4.5
4.0
3.5
3.0
2.5
2.0
1.5
1.0
0.5

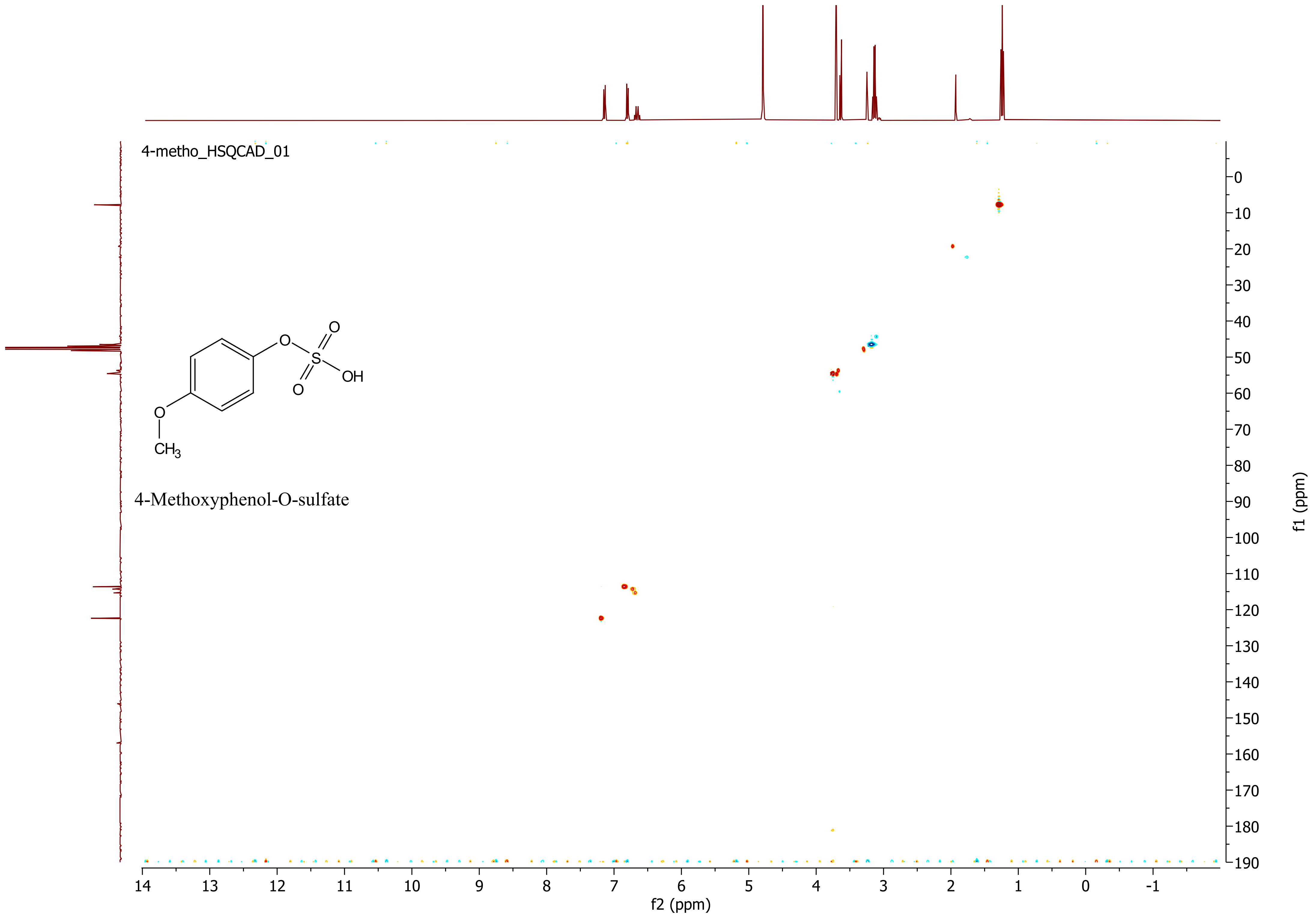

4-metho_HSQCAD_01
O
S
O
O
OH
O
CH3
4-Methoxyphenol-O-sulfate
f1 (ppm)
f2 (ppm)

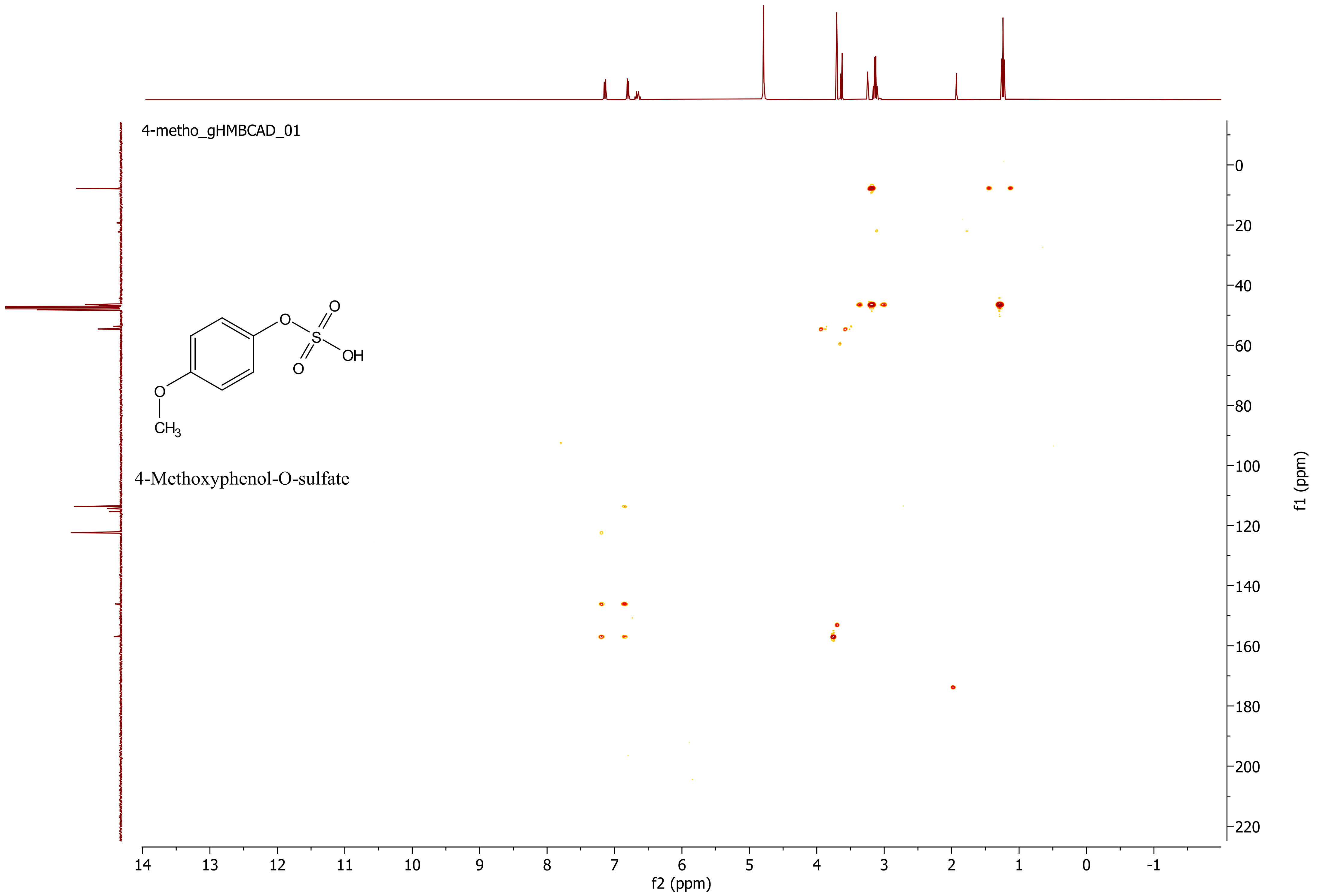
4-metho_gHMBCAD_01
4-Methoxyphenol-O-sulfate
O
S
OH
CH3
f2 (ppm)
f1 (ppm)
14
13
12
11
10
9
8
7
6
5
4
3
2
1
0
-1
0
20
40
60
80
100
120
140
160
180
200
220
